# Audio Recording Patient-Nurse Verbal Communications in Home Health Care Settings: Pilot Feasibility and Usability Study

**DOI:** 10.2196/35325

**Published:** 2022-05-11

**Authors:** Maryam Zolnoori, Sasha Vergez, Zoran Kostic, Siddhartha Reddy Jonnalagadda, Margaret V McDonald, Kathryn K H Bowles, Maxim Topaz

**Affiliations:** 1 School of Nursing Columbia University New York, NY United States; 2 Center for Home Care Policy & Research Visiting Nurse Service of New York New York, NY United States; 3 Electrical Engineering Columbia University New York, NY United States; 4 Amazon Seattle, WA United States; 5 School of Nursing University of Pennsylvania Philadelphia, NY United States

**Keywords:** patients, HHC, communications, nurse, audio recording, device

## Abstract

**Background:**

Patients’ spontaneous speech can act as a biomarker for identifying pathological entities, such as mental illness. Despite this potential, audio recording patients’ spontaneous speech is not part of clinical workflows, and health care organizations often do not have dedicated policies regarding the audio recording of clinical encounters. No previous studies have investigated the best practical approach for integrating audio recording of patient-clinician encounters into clinical workflows, particularly in the home health care (HHC) setting.

**Objective:**

This study aimed to evaluate the functionality and usability of several audio-recording devices for the audio recording of patient-nurse verbal communications in the HHC settings and elicit HHC stakeholder (patients and nurses) perspectives about the facilitators of and barriers to integrating audio recordings into clinical workflows.

**Methods:**

This study was conducted at a large urban HHC agency located in New York, United States. We evaluated the usability and functionality of 7 audio-recording devices in a laboratory (controlled) setting. A total of 3 devices—Saramonic Blink500, Sony ICD-TX6, and Black Vox 365—were further evaluated in a clinical setting (patients’ homes) by HHC nurses who completed the System Usability Scale questionnaire and participated in a short, structured interview to elicit feedback about each device. We also evaluated the accuracy of the automatic transcription of audio-recorded encounters for the 3 devices using the Amazon Web Service Transcribe. Word error rate was used to measure the accuracy of automated speech transcription. To understand the facilitators of and barriers to integrating audio recording of encounters into clinical workflows, we conducted semistructured interviews with 3 HHC nurses and 10 HHC patients. Thematic analysis was used to analyze the transcribed interviews.

**Results:**

Saramonic Blink500 received the best overall evaluation score. The System Usability Scale score and word error rate for Saramonic Blink500 were 65% and 26%, respectively, and nurses found it easier to approach patients using this device than with the other 2 devices. Overall, patients found the process of audio recording to be satisfactory and convenient, with minimal impact on their communication with nurses. Although, in general, nurses also found the process easy to learn and satisfactory, they suggested that the audio recording of HHC encounters can affect their communication patterns. In addition, nurses were not aware of the potential to use audio-recorded encounters to improve health care services. Nurses also indicated that they would need to involve their managers to determine how audio recordings could be integrated into their clinical workflows and for any ongoing use of audio recordings during patient care management.

**Conclusions:**

This study established the feasibility of audio recording HHC patient-nurse encounters. Training HHC nurses about the importance of the audio-recording process and the support of clinical managers are essential factors for successful implementation.

## Introduction

Patients’ spoken language provides a window into a wide range of pathological entities, including pulmonary hypertension [1], respiratory obstruction [2], neurological disorders [3], and mental illnesses [4], enabling spoken language to act as a biomarker for screening patients with these diseases and symptoms. Recently, emergent studies have used established procedures in phonetics, speech sciences, and natural language processing to estimate changes in the phonatory and articulation of the patient’s voice and to analyze semantic and pragmatic levels of language organization; these studies developed diagnostic and risk identification algorithms for the timely detection of diseases, particularly neurological and mental disorders [3,4].

Despite the promising findings of these studies, the audio recording of patients’ spontaneous speech is not a part of routine clinical workflows. Most studies on patients’ speech were cross-sectional and conducted in laboratory (controlled) settings, where patients were instructed to follow specific diagnostic and screening tests (eg, verbal fluency test and describing a positive or negative emotion) without having any interaction with clinicians [5-8]. These studies have several limitations, particularly in design and small sample sizes, which in turn limit the performance and generalizability of diagnosis or risk identification algorithms [3,4]. Integrating audio recordings of patient-clinician verbal communications during routine clinical encounters can potentially help resolve these limitations by creating an analytic pipeline of data sets to model subtle changes in patients’ language, voice, emotion, interaction patterns, and engagement during clinical encounters. Such recordings can serve as a basis for developing intelligent clinical decision support systems that can help diagnose medical conditions or identify patients at risk for deterioration and negative outcomes.

Health care stakeholders’ perspectives toward audio recording patient-clinician verbal communication have been discussed in previous studies. A recent study that investigated policies for audio recording patient-clinician encounters in the 49 largest health care systems in the United States found that despite physicians’ willingness to audio record patient-clinician encounters, none of the health care systems had a dedicated policy or guidance for integrating audio recordings of patient-clinician encounters into clinical workflow [9]. In another study, Meeusen et al [10] explored patients’ perspectives on the recording of their communication with neurosurgeons in an outpatient setting. Overall, patients had a positive perspective on recording, and they found it helpful. They also recommended that their future communication with clinicians be recorded [10]. In addition, Ball et al [11] also evaluated the perspectives of patients, clinicians, and clinic leaders toward audio recording patient-clinician encounters. The findings showed that patients saw audio recording as an opportunity to improve care. However, clinicians found it disruptive and burdensome but appreciated its value for receiving low-stakes constructive feedback. Clinic leaders had a positive perspective on recording but were not prepared for its implementation in the clinical setting [11].

There is a growing consensus on the usability of home health care (HHC) technology as a significant factor affecting the use of technology in the HHC settings. HHC is a health care setting where services are provided by skilled practitioners (often registered nurses) to patients in their homes [12]. HHC patients are often clinically complex and vulnerable as they are generally older adults aged ≥65 years, with multiple chronic conditions such as Alzheimer disease and related disorders and respiratory and cardiac diseases. They are also at risk for negative outcomes such as emergency department visits and hospitalizations [13,14]. Audio recording and modeling of HHC patient-nurse encounters can allow the HHC team to enrich the documentation of patients’ information in electronic health records (EHRs) and facilitate the development of high-performing clinical decision support systems to identify HHC patients at risk of health care deterioration (eg, Alzheimer disease), communication deficits (eg, aphasia), and negative outcomes (eg, emergency department visit). To assess the usability of technology such as audio recording of patient-nurse verbal communication in HHC settings, the International Organization for Standardization introduced 3 metrics: effectiveness, efficiency, and satisfaction [15]. Effectiveness mainly measures the extent to which the technology achieves its intended clinical goal, such as improvement in the performance of a diagnostic algorithm in diagnosing Alzheimer disease. Efficiency is related to the time and physical or mental effort needed to accomplish a task. Satisfaction measures the perceived usefulness and ease of use of technology from the perspective of health care stakeholders, including clinicians and patients. The Association for the Advancement of Medical Instrumentation and the Human Factors and Ergonomics Society emphasized the importance of HHC stakeholders’ satisfaction and the conduction of a human factor analysis to improve usability [16]. HHC technology may be proven effective and efficient from the perspective of developers and researchers; however, HHC stakeholders, particularly patients and nurses, may find it unsatisfactory because of human factor issues such as substation mental efforts or the time needed to learn about the technology.

Although some studies have reported on audio recording of clinical encounters mainly for patients’ personal use (eg, recall of visit information) [17,18], few published insights are available on the usability of audio-recording devices and the accuracy of the recorded verbal communications. Most audio-recording devices can provide a sufficient level of quality for patients’ personal use and documentation purposes; however, they do not offer the required accuracy for modeling the properties of a patient’s verbal communication, particularly vocal (acoustic) parameters. In addition, the quality of recorded communication is highly dependent on the context of the clinical setting. In clinical settings where patients and clinicians need to constantly move for physical examination or therapy, such as HHC settings [12], the location of the audio-recording device and the background noise can significantly affect the quality of the recorded communication and, in turn, the modeling verbal communication parameters. To integrate audio recordings into clinical workflows, it is also critical to consider patient and clinician attitudes and concerns regarding the audio-recording process. A patient’s or clinician’s negative attitude or discomfort during the recording process may disrupt the flow of treatment or result in the Hawthorne effect [19], affecting clinicians’ communication patterns and treatment practices and preventing patients from sharing their actual concerns with clinicians.

To address the gaps in the literature, this study aimed to (1) evaluate the functionality and usability of several audio-recording devices for audio recording patient-nurse verbal communication in the HHC setting and (2) elicit the perspectives of HHC stakeholders (patients and nurses) about the facilitators of and barriers to integrating audio recording into clinical workflows.

## Methods

### Study Setting

**Figure 1:**
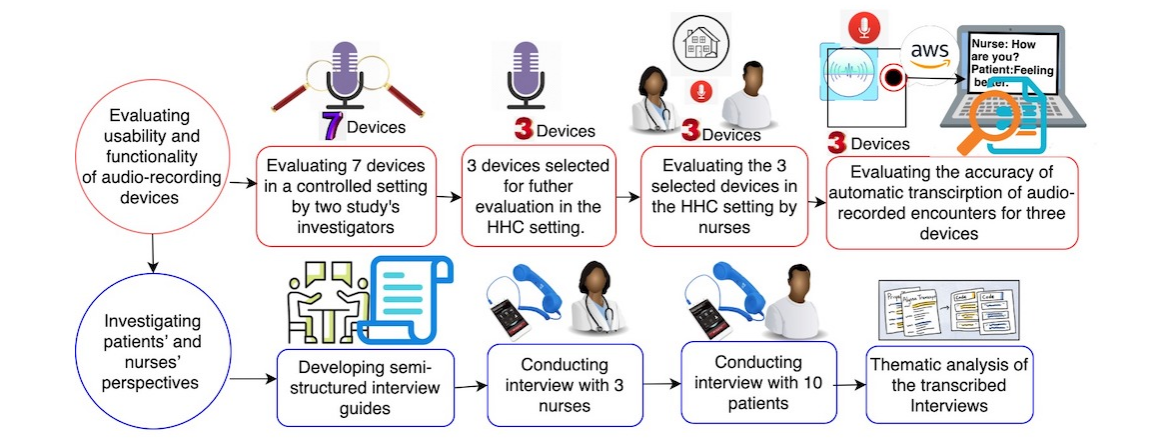
A schematic view of the methodology of the study. HHC: home health care.

This descriptive feasibility study was conducted at the largest not-for-profit home health agency in the United States. The agency has approximately 10,600 staff, including 1470 nurses and >6500 home health aides. In 2019, the agency served >106,000 unique patients across >1.08 million clinical visits. A summary of patients’ demographic information in 2019 showed that most of the Visiting Nurse Service of New York (VNSNY) patients were aged >65 years, predominantly women (63483/106,000, 59.89%), and almost half were African American or Hispanic (25,779/106,000, 24.32%, and 23,139/106,000, 21.83%, respectively). Figure 1 provides a schematic of the methodology used in this study.

### Ethics Approval

This study was approved by the Visiting Nurse Service of New York Institutional Review Board (reference #E20-003).

### Evaluating the Functionality and Usability of Audio-Recording Devices

#### Overview

**Figure 2:**
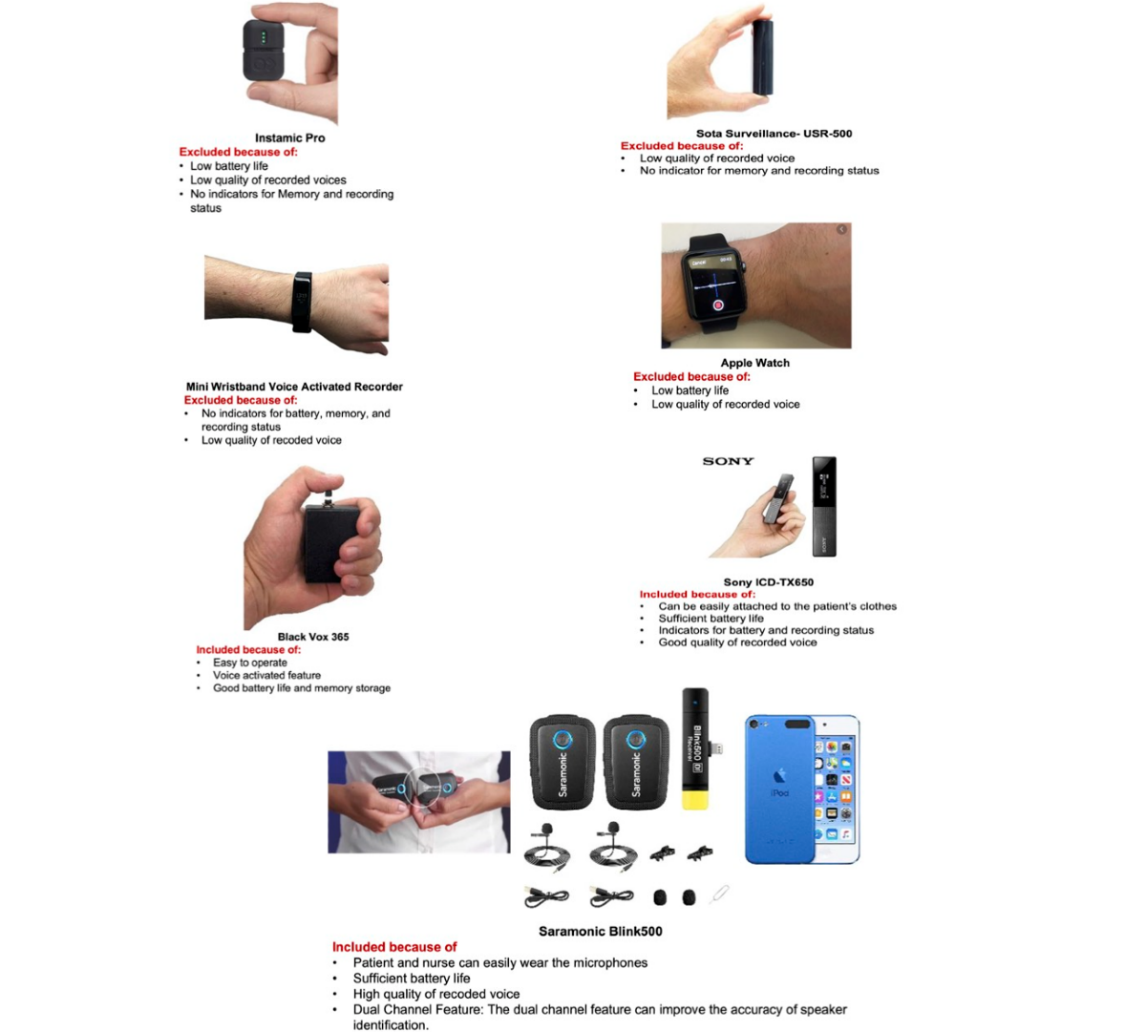
Audio-recording devices evaluated for audio recording of patient-nurse verbal communication.

In the first phase of the study, we created a list of criteria for selecting the audio-recording devices. The criteria included the portability of the device, wearable features, functionality of the device (memory size and battery life), and voice activation features. We reviewed the features of >50 audio-recording devices from different web-based sellers, such as Amazon, BestBuy, and SpyCenter. We selected 7 devices that met the criteria for the audio-recording device for the study. We evaluated the 7 devices for audio recording HHC patient-nurse verbal communication. All the devices were portable, with relatively simple operation, and could be used easily for recording verbal communication without disrupting the participants’ movements during communication. The devices used were Black Vox 365, SOTA Surveillance-USR500, INSTAMIC PRO, Sony ICD-TX6, Mini Wristband Voice Activated Recorder, Apple Watch, and Saramonic Blink500 Pro B2. Figure 2 shows images of the audio-recording devices.

#### Controlled Setting

In the first phase of evaluation, 2 study investigators (MZ and SV) used the 7 devices to audio record verbal communications between themselves in a controlled environment, which resembled the verbal communication between patients and nurses in HHC settings. The investigators evaluated the devices using 8 criteria: memory size; battery life; indicators for memory size, battery life, or recording status; automated voice activation feature; ease of device attachment to clothing; and accuracy of automatic transcription of recorded verbal communications as an indicator of voice quality (see the *Transcription Accuracy* section for evaluation metrics of automated voice activation features). The SOTA Surveillance-USR500, INSTAMIC PRO, Mini Wristband Voice Activated Recorder, and Apple Watch were excluded from further evaluation in the clinical settings because of their low battery life; low memory capacity; no indicators for recording (on or off), battery, and memory status; and low accuracy of automatic transcription of recorded communications. A total of 3 recorders, Black Vox 365 (further referred to as Vox), Sony ICD-TX6 (further referred to as Sony), and Saramonic Blink500 Pro B2 Pro (further referred to as Saramonic), were included for further exploration in HHC because of the relatively high accuracy of automatic transcription, easy operation, good battery life, and memory capacity.

The Saramonic device comprises 2 wireless microphones that transmit the captured audio to a receiver connected to a recording device such as an iPod. The device has the feature of recording the communication between 2 people (eg, patient and nurse into the 2-channel recording), which can facilitate the process of separating the patient’s voice from the nurse’s voice for analysis purposes. By contrast, Sony and Vox embedded microphones with single-channel features. In contrast with Vox, Sony and Saramonic could be easily attached to the patients’ and nurses’ clothes and included indicators for recording status (on or off). Vox had a voice activation feature that was not available in Saramonic and Sony.

#### HHC Setting and Participant Recruitment

Next, we collaborated with 2 HHC nurses who used the 3 selected devices to audio record their verbal communication with patients during HHC encounters. With HHC organizational support of the study and after institutional review board approval, we approached nurses through email and advertising at the VNSNY site. Interested nurses provided written consent. The consent form included information about the aim of the study and its potential risks and benefits. A research assistant (RA) trained the nurses to use the audio-recording devices.

Several different strategies were used to engage patient participants. The first strategy involved nurses providing flyers to patients with a brief description of the study. Nurses provided the RA with the name and contact information of patients who expressed interest. The second strategy was that after a waiver of authorization was granted, the RA reviewed the nurse’s schedule in the EHR to identify patients on the nurse’s caseload. The RA contacted the potential patient participants by phone and described the study’s aim, potential risks, and benefits. For those who provided verbal consent, the RA mailed the consent form to the patients for their reference. Both nurse and patient participants received a gift card as a token of appreciation.

The participating nurses audio recorded their routine HHC encounters with consented patients. Audio-recorded encounters were uploaded to a secure server. Nurses also completed the System Usability Scale (SUS) questionnaires [20] for each device. The SUS is a robust and reliable instrument that measures a product’s usability from the user’s perspective. An example statement is, “I thought the system was easy to use.” The SUS provides scores from 0 (negative) to 100 (positive), with a standard average score of 68. A score >80 indicates excellent usability, whereas a score <68 indicates that the product has usability issues that are a cause for concern. To further evaluate the usability and functionality of the devices, we conducted semistructured, open-ended interviews with nurses to collect their opinions about the functionality and usability of the devices.

### Transcription Accuracy

The quality of audio-recorded communications affects the transcription accuracy of a specific automatic speech recognition system such as the Amazon Web Service General Transcribe System (AWS-GTS) [21]. For example, the background noise and volume of the captured voice affect the quality of the transcription. We quantified the quality of audio recording patient-nurse verbal communications for the 3 devices using the automatic transcription and speaker identification features of the AWS-GTS. AWS-GTS was built on deep neural network models and trained on a large body of labeled (manually transcribed) verbal communication. We used this system as it is Health Insurance Portability and Accountability Act compliant and currently in use at the participating HHC organization.

We evaluated 2 components of transcription quality, word error rate (WER) and speaker identification accuracy, using the steps described in Textbox 1.

**Transcription quality evaluation steps.**

**Step 1**

For each device (Vox, Sony, and Saramonic), we randomly selected 3 audio-recorded home health care encounters.

**Step 2**

One of the investigators transcribed the audio files manually and assigned each utterance (defined as the uninterrupted part of the dialog expressed by one of the speakers) to an appropriate speaker (patient or nurse). Manual transcription was reviewed by a second investigator to ensure the quality of the transcription.

**Step 3**

All audio files were transcribed using the Amazon Web Service General Transcribe System application programming interface. The application programming interface returns the transcriptions as JSON files, including the start and end times of each transcribed word and the assigned speaker (eg, speaker 1 vs speaker 2) to each word. The transcribed words were joined to form an utterance using the type of speaker and converted to a Microsoft Excel sheet. We define an “utterance” as a continuous block of the uninterrupted speech of a single speaker.

**Step 4**

The quality of automatic transcriptions at the utterance level was compared with manual transcription and measured using the word error rate (WER). WER is a common metric for measuring the performance of speech recognition systems. It is computed based on the number of substitutions, insertions, and deletions that occur in a sequence of recognized words using a speech recognition system. The WER score starts from 0 (indicating no error in transcription) and can reach any score >1 depending on the length (number of words) in the utterance or document. For comparison, the average WER for human transcriptions is 0.04 (4%). Our earlier preliminary study for measuring the quality of transcription of an open-source automatic speech recognition system, Wav2Vec, on a subset of audio-recorded patient-nurse encounters provided a WER of 0.98 (98%). Wav2Vec is an unsupervised pretraining for speech recognition that learns representations of raw audio and was developed by the Facebook Company [22].

Second, we used the speaker identification feature of AWS-GTS to measure the accuracy of the automatic transcription of audio-recorded patient-nurse verbal communication by the devices. We expected that multiple-channel audio-recording devices (ie, Saramonic) would provide higher accuracy for speaker identification than that provided by single-channel devices (ie, Vox and Sony). To measure the accuracy of speaker identification, we used the following steps:

- Step 1: All manually transcribed audio files with assigned speakers at the utterance level (patient or nurse) were tokenized into words, and each word was linked to the assigned speaker.
- Step 2: The words of each manually transcribed utterance were mapped to the corresponding utterances and words provided by AWS-GTS.
- Step 3: We computed the percentage of words with accurate speaker identification with references to the total number of transcribed words in each audio file.

### Investigating Patients’ and Nurses’ Perspectives About the Facilitators of and Barriers to the Integration of Audio-Recording of Verbal Communications Into HHC Clinical Workflows

#### Developing Semistructured Interview Guidelines

To understand the facilitators and barriers to the integration of audio recording of patient-nurse encounters into the clinical workflow, we conducted semistructured interviews with patients and nurses. The questions for nurses mainly covered their experience, concerns (eg, Hawthorne effect), potential benefits of recording (for both patients and clinicians), and their overall attitude toward the integration of audio-recording processing into HHC clinical workflows. The questions for patients covered their motivation to participate in the study, their concerns, and their attitudes toward audio recording their conversations with nurses. All the research questions were reviewed and discussed by the research team, 2 nurses with expertise in HHC services, a patient representative (familiar with HHC patients’ characteristics, needs, preferences, and concerns), and 2 health informaticians. The study team ensured that the topics of the semistructured interviews would lead to the discovery of major facilitators and barriers to the development of a practical approach for audio recording patient-nurse encounters in a clinical setting. The questions for the semistructured interviews are presented in Multimedia Appendix 1. Results of the interview guide development team discussions were summarized to generate an initial codebook for thematic analysis of the interviews.

#### Engagement of Study Participants in Qualitative Interviews

A total of 5 nurses audio recorded their communication with patients. However, during the study period, 40% (2/5) of nurses left the participating agency and were therefore not available to participate in the interviews. The nurses participated in audio-recorded encounters with 45 patients in this ongoing study. After securing additional consent, we conducted interviews with the remaining 60% (3/5) of nurses.

To reduce the likelihood of selection bias in creating a sample of HHC patients for the interviews, we used a stratified sampling technique. Using this technique, we stratified the pool of patients who participated in the audio recording of patient-nurse verbal communication (45 patients) based on the study nurse who recorded the encounter. Next, from each group of patients, the RA randomly contacted 2 patients for interviews. If the patient agreed to participate, the RA consented the patient for the study. This process was repeated until data saturation was achieved by interviewing a sample of 22% (10/45) of HHC patients.

To address the patient’s potential concern about privacy and confidentiality of the information collected during the interviews and ensure that the patient expressed their unbiased perspectives, our RA consented the patients by explaining our commitment and strategies to protect the patients’ privacy and confidentiality. In addition, patients were informed that they could withdraw from the study (audio recording of patient-nurse verbal communication) without any consequences and had the freedom not to answer any questions.

In addition, our RA, who conducted the interviews, was well trained for effective communication (eg, active listening without interruption and speaking slowly and clearly) with VNSNY’s patient population and had extensive experience in interviewing HHC patients for different qualitative studies. Gift cards were offered to both nurses and patients for participating in the qualitative interviews.

#### Thematic Analysis of the Interviews

The team created an initial codebook to summarize the open-ended interviews. Next, we used a thematic analysis approach for the systematic coding of the interviews. Thematic analysis is a qualitative descriptive approach for identifying, analyzing, and reporting themes within data [23-25]. The analysis phases were (1) familiarization with data, (2) generating initial codes, (3) data coding, (4) intercoder reliability, (5) searching for themes, (6) defining and naming themes, and (7) producing the report. To familiarize them with the data (step 1), 2 team investigators listened to and reviewed the transcribed interviews and used the initial codebook (step 2) to code transcriptions using a Microsoft Excel spreadsheet (step 3). The codes were assigned to informative passages of transcriptions (phrases, sentences, or paragraphs). Intercoding reliability was assured by dual coding of the first 2 interviews for each stakeholder (patient and nurse). Disagreements were resolved through discussions between the coders. Next, each investigator continued and coded the remaining interviews separately. When new codes emerged, they were discussed in our regular team meeting and added to the coding scheme if necessary (step 4). Final codes were collated into potential themes (broader and more abstract than codes) by undertaking an interpretative data analysis (step 5). The themes that emerged were further refined by analyzing the aspects of the data captured by each theme (step 6). This helped us to generate informative and clear names and definitions for a theme. The final report was produced by linking each theme to vivid and compelling interview quotes. Analysis of the patients’ interviews and patient selection proceeded in an iterative process until data saturation was achieved (no new themes emerged from the data, and each theme was refined within a diverse sample).

## Results

### Demographic Information

Table 1 includes a summary of demographic information of the patients who participated in this study. The patients were predominantly men (6/10, 60%). Approximately 40% (4/10) of the study participants were Black, 30% (3/10) were White, and 20% (2/10) were Hispanic. One of the patients was not interested in declaring their race. More than half of the patients (6/10, 60%) were retired, and some (2/10, 20%) patients were disabled. All 3 nurses participating in this study were women: 2 (67%) were Black, and 1 (33%) was Hispanic. All 3 nurses had >5 years of clinical experience in HHC settings.

**Table 1:** Demographic information of patients (n=10) and nurses (n=3) participating in this study.

Demographics				Participants
**Patients**				
	Gender (female), n (%)		4 (40)	
	**Ethnicity, n (%)**			
		Black	4 (40)	
		White	3 (30)	
		Hispanic	2 (20)	
		Other	1 (10)	
	Age (years), mean (SD)		59.7 (16.25)	
	**Employment, n (%)**			
		Employed	1 (10)	
		Unemployed	1 (10)	
		Retired	6 (60)	
		Disabled	2 (20)	
**Nurses**				
	Gender (female), n (%)		3 (100)	
	**Ethnicity, n (%)**			
		Black	2 (67)	
		White	—^a^	
		Hispanic	1 (33)	
		Other	—	
	**Work experience (years), n (%)**			
		<5	0 (0)	
		5-10	2 (67)	
		>10	1 (33)	

^a^None of the nurses participated in this study was White.

### Usability

Sony had the highest SUS score compared with Vox and Saramonic. Although the Saramonic device had a slightly lower SUS score than the SUS score of the Sony device, nurses found Saramonic easier in terms of approaching HHC patients for permission to audio record the verbal communication because of the appearance of the device and flexibility in attaching the microphone to the patient’s clothes. In addition, the quality of audio-recorded communication using Saramonic was higher than that of the other 2 devices when measured using the WER of automatic transcription and accuracy of speaker identification provided by AWS-GTS. As expected, the desirable usability feature of Vox was automatic voice-activated recording. However, this feature might compromise the patient’s or nurse’s privacy if the nurse forgets to pick up the device from the patient’s home or if the nurse forgets to turn off the device, which in turn would start recording unrelated conversations. This feature was not available for Sony or Saramonic. Overall, we found that Saramonic is the most appropriate device for recording patient-nurse encounters with the highest SUS score and accuracy of speaker identification, as shown in Table 2.

**Table 2:** Evaluation of 3 audio-recording devices in home health care settings by nurses and quality of audio-recorded files measured using Amazon Web Service General Transcribe System.

Device	System Usability Scale score	Overall opinion of the nurses about the device	Word error rate (%)	Accuracy of speaker identification (%)
Vox	42.5	The device lacked ease of usability because of the lack of indication of both the battery life and recording status.	38.4	67.3
Sony	78.75	The device was lightweight and easy to use; however, with the ongoing COVID-19 pandemic, the nurses were not comfortable putting the microphones near the face.	27	89.6
Saramonic	65	The device was simple to use. In addition, it was easier to approach patients using this device than with Sony.	26.3	91.3

### Stakeholder Perspectives

We investigated the perspectives of HHC stakeholders (patients and nurses) toward integrating the audio-recording of patient-nurse verbal communications into the clinical workflow. Table 3 provides the thematic analysis of the interviews with patients. Overall, most patients were comfortable with the procedure of audio recording their communication with the nurses. Some patients even mentioned that they completely forgot that their communication with the nurse was being recorded, and they did not have any concerns about sharing their concerns with nurses. In addition, most patients perceived the potential benefits of audio recording, particularly as a mechanism for recalling the nurses’ instructions, and they wanted the audio-recorded files to be shared with them.

**Table 3:** Summary of patients’ interviews.

Themes and subthemes		Common findings across patients	Differences across patients
Reasons for study enrollment		Most patients stated that their primary reasons for enrollment were to potentially help others and as they were satisfied with the services their nurses had provided them.	A patient had stated their primary reason for enrollment was the financial incentive.
**Experience with recording**			
	Perceptions	Most patients had expressed feeling confident and that the device was not bothersome and was comfortable. Multiple patients stated that they forgot about the presence of the recorder soon after the visit started.	—^a^
	Communication	All patients expressed that there was no effect on their communication with the nurses.	—

^a^There were no differences on the perceptions of nurses participated in this study.

Table 4 presents a summary of the thematic analysis of the nurses’ interviews. All nurses agreed that the recording device was convenient and easy to use, and they became used to the procedure after audio recording a few encounters (2-3 encounters each). However, some nurses disputed the idea of integrating the audio-recording of patient-nurse encounters into HHC clinical workflows because of the lengthening of the duration of the encounters, which cannot be easily suited to their current heavy workloads. For example, one of the nurses mentioned that “I don’t know. We are so busy. I remember there was a day I had 10 plus patients, that day I did not take the recorder. I don’t have time to do that.” When we investigated nurses’ opinions about the usefulness of audio-recorded verbal communication, some of them expressed doubt and argued that all the patient health–related information is documented in the EHR system, and audio recording probably would not add more information. A nurse found the recording process helpful for managers in gaining insights into the quality of care in HHC settings. When asked about the impact of the audio recording on patient-nurse communication patterns, nurses stated that the recording would likely affect their communication patterns with patients. Specifically, audio recording may increase nurses’ willingness to establish more formal relationships with patients. Nurses also believed that patients’ communication patterns would change in the presence of audio-recording devices. When asked about the patients’ willingness to participate in this study, they responded that it depended on the patient’s personality, proactiveness in their care, and the complexity of their medical conditions.

**Table 4:** Summary of nurses’ interviews.

Themes and subthemes		Common findings across nurses	Differences across nurses
**Experience with recording**			
	Device usability	Most nurses had a good experience and stated that they began to become comfortable after a few uses (recording 1-2 encounters).	A nurse highlighted the difficulty in finding places to clip the microphone and attach the device in one home health care encounter.
	Future use	When asked if this would become a standardized process, most nurses expressed dismay, citing that it would add more time to the visits with their already heavy workload.	—^a^
	Potential use of recording for other purposes	Most nurses expressed doubts about its usefulness because of their present, heavy workload and no need to use it to look back at visits as everything is documented during the visit.	A nurse expressed the usefulness in a management aspect where it can potentially help with the assignment of the workload. It would help give insights into the visits that they would not otherwise see.
**Patient encounters**			
	Communication	Most nurses said that the recording could affect the way patients communicate with nurses (Hawthorne effect). Most nurses said that the recording could affect the way the nurses develop relationships with their patients. In other words, it may increase the nurses’ willingness to establish a more formal relationship with patients.	—
	Participation	—	All the nurses answered differently. It could be dependent on the patients’ personality, proactiveness in their care, or the type of case they are in (chronic care or wound care).

^a^There was no difference across nurses.

## Discussion

### Principal Findings

This study is the first to develop a practical approach for audio recording HHC patient-nurse verbal communication and evaluate the feasibility of integrating this approach into the clinical workflow. We showed that the type of recording device can have a differential effect on the accuracy of downstream tasks built on audio-recorded patient-nurse encounters, such as automatic transcription and provided speaker identification. In addition, our results suggest that HHC stakeholders’ attitudes toward the recording process are a crucial factor affecting the successful integration of the audio recording of patient-nurse verbal communications into the clinical workflow.

Selecting an appropriate recording device is of great importance to designing and implementing an effective approach for audio recording clinical encounters in HHC settings. Some devices are lightweight, can be easily attached to the participants’ clothes, and have a very simple operation mechanism with one on or off button. However, these devices often have functionality issues, such as short battery life or limited memory size, which reduce their usability by increasing the number of clinicians or administrative staff. The INSTAMIC PRO device is an example of such a device with high ease of use but low functionality because of its small memory capacity. In contrast, some devices are easy to use with good functionality; however, because of their size or weight, they cannot be easily attached to a patient’s or clinician’s clothes. Vox is an example of a device that needs to be set on a flat surface (eg, a table) during the process of recording. Although this solution is practical for recording patient-clinician encounters where no movement is required during communication, it is not an appropriate solution in HHC settings where there is a constant required movement of the patient or nurse for physical examination or treatment. The patient’s or nurse’s movement will change their position with reference to the device location and, therefore, would affect the quality of audio-recorded communication, in turn affecting downstream tasks built on the audio data (eg, extracting some linguistic or acoustic features from the patient’s speech).

In addition, the quality of the audio-recorded communication by devices substantially affects the quality of automatic transcription and speaker identification provided by an automatic speech recognition system. This is particularly important when the goal of the study is to develop an automatic analytic pipeline for processing and modeling patient-nurse verbal communication to develop a risk identification or diagnostic algorithm (eg, a diagnostic algorithm for Alzheimer disease). Regarding the possibility of background noise in HHC settings, which comes from different sources such as television, air conditioner, or a caregiver’s speaking, it is important to set the microphone as close to the patient’s and nurse’s mouths to reduce the possibility of background noise captured by the device’s microphone. An option for reducing background noise is to use a unidirectional (cardioid) overhead microphone; however, as this study was conducted during the COVID-19 pandemic and the study participants were recognized as being at high risk for COVID-19, we avoided any devices that touched the patient’s face. Unidirectional microphones pick up audio (eg, the patient’s voice) from only the front compared with omnidirectional microphones that pick up audio from all directions. Another reason that convinced us not to use overhead microphones was the risk of patient discomfort during the recording procedure when the microphone touches the patient’s face.

Another important feature of audio-recording devices is the number of channels with the ability to separate audio tracks for each individual participating in the communication. Devices with this type of feature usually have multiple microphones that are used by several individual speakers. The voice captured by each microphone is transmitted to an individual channel. This feature is important for downstream tasks, especially the differentiation of the patient’s voice from the nurse’s voice. Among all the devices evaluated in this study, the Saramonic device was the only device that included this feature; consequently, it had a higher score for speaker identification when it was measured using AWS-GTS. Overall, because of the better quality of automatic transcription and speaker identification (measured using AWS-GTS) of verbal communications recorded by this device and the high score for usability, we selected this device for audio recording further encounters beyond the pilot assessment.

Understanding HHC patients’ and nurses’ perspectives toward audio recording is a key determinant of the successful integration of the audio recording of patient-nurse verbal communication into the HHC clinical workflow. Overall, patients found the process of audio recording to be satisfactory and convenient, with minimal impact on their communication with nurses. Patients expressed that they were able to freely share their concerns and health care issues with nurses, and they even forgot about the presence of audio devices soon after the visit started. This expression implies that the Hawthorne effect on patients’ communication patterns with nurses was minimal. It also shows the practicality of the recording procedure designed for this study.

The Hawthorne effect refers to a study participant’s reactivity, in which the participant changes an aspect of their behavior in response to their awareness of being observed by the study’s investigator [19]. At the beginning of the study, we briefly educated the patients about the audio-recording procedure and our strategy for protecting their privacy and confidentiality. We believe that this training is particularly essential for reducing the possibility of patient discomfort and, in turn, the Hawthorne effect.

Similar to patients, nurses found the audio-recording procedure satisfactory and easy to learn. However, in contrast to patients, nurses expressed that audio recording of HHC encounters can affect their practice and communication patterns in the HHC settings. Some nurses suggested that their communication with patients might become more *formal* rather than personal. Therefore, we may conclude that the presence of a recording device can introduce a Hawthorne effect on nurses’ communication with patients. Nurses expressed concern about sharing audio-recorded encounters with supervisors for evaluation purposes. Owing to this concern, we experienced difficulties in recruiting nurses to audio record their communication with patients; however, after educating nurses about protecting their privacy and confidentiality, 5 nurses agreed to participate in the study. Nurses were not very optimistic about the usability of integrating audio recording in clinical workflows as they perceived limited benefits in audio recording the HHC encounters. This is contrary to the findings of numerous studies [1-8] showing the importance of patient-spoken language in the development of diagnostic and risk identification algorithms for identifying patients with pathological entities and at risk of negative outcomes. Educating nurses about the importance of patients’ spoken language may partially resolve this problem. In addition, nurses had concerns about increasing the length of HHC encounters because of the time required to set up the audio-recording device, which was a challenge given their existing heavy workload. The use of devices that are minimally burdensome and easy to use and enlisting the support of clinical managers are essential to address these issues and are key for successful integration.

### Limitations

The findings of this study should be considered in light of several limitations. First, although the audio-recording devices selected for this study included a wide range of useful and convenient features for audio recording HHC patient-nurse encounters, they may not represent all features of existing devices in the market. For example, future studies may investigate the usability and functionality of Amazon Alexa (a device that was developed by the Amazon company) for audio recording patient-nurse encounters, the possibility of connecting to a secure server for storing the audio-recorded data, and the potential risks to patient privacy and confidentiality. Second, we quantified the quality of audio-recorded communication by measuring the accuracy of the automated transcription provided by AWS-GTS. Although the quality of audio-recorded data is correlated with the accuracy of automated transcription, it may not provide comprehensive insights into the quality of audio recorded by a device. Future studies may investigate other measures such as the sensitivity of the device for filtering background noise in noisy clinical settings to better evaluate the quality of the audio-recorded voice by the device. Third, as this study was conducted during the COVID-19 pandemic, recruitment and retention of HHC nurses were challenging. This was mostly because of nurses’ heavy workload, and precautions needed to be taken to reduce the risk of COVID-19 transmission in the HHC setting. In addition, because of the COVID-19 pandemic, it was challenging for the research team to reach out to all nurses with a potential interest in participating in this study. Overall, we were able to recruit 5 nurses for the study of audio recording patient-nurse verbal communication conducted at VNSNY. Of the 5 nurses, 2 (40%) nurses left the VNSNY during the study. Hence, unfortunately, we could not reach the 2 nurses to solicit their perspectives. Although a sample size of 3 nurses may not provide sufficient data to achieve data saturation, the 3 nurses who participated in the interviews had extensive experience in HHC services, HHC workflow, and working with a racially diverse patient population in VNSNY. Therefore, they were able to provide a valuable evaluation of the facilitators and barriers to the pipeline designed for audio recording and its integration into the HHC workflow. Aggregation of the 3 nurses’ perspectives sheds light on the facilitators and barriers to a large extent and was very informative for HHC managers and policy makers. For example, the manager of the VNSNY research center informed us that they were willing to address some of the barriers (eg, training nurses about the importance of audio-recording encounters) to encourage more HHC nurses to participate in similar studies in the future. Currently, we are actively recruiting more nurses for this study, which will enable us to provide a better picture of nurses’ opinions toward this process in our future report. Fourth, the racially diverse sample size of patients (10/45, 22%) who participated in this study was achieved through an iterative process and data saturation of thematic analysis of interview findings, which represents the patient population at the study agency to a large extent. However, the findings regarding patients’ opinions may not provide a deep insight into the attitudes of ethnic minority patients or those with complex clinical conditions. Finally, this study was conducted at one agency, the largest HHC organization in the United States. However, there might be differences in the types of health care services, communication, and practice patterns across HHC. Therefore, the overall findings of this study may not be representative of all HHC settings.

### Conclusions

To develop an effective practical approach for integrating audio recording of patient-nurse verbal communication in HHC settings, it is essential to select an audio-recording device with high functionality and usability. Training nurses and clinical managers on the importance of audio-recorded verbal communication can encourage them to support the process of integration.

In addition, training can reduce the potential concerns of nurses about protecting their privacy and confidentiality during the recording process.

## Appendix

Multimedia Appendix 1 Questions for semistructured interviews.

## Abbreviations

AWS-GTS: Amazon Web Service General Transcribe System
EHR: electronic health record
HHC: home health care
RA: research assistant
SUS: System Usability Scale
VNSNY: Visiting Nurse Service of New York
WER: word error rate
