# Supplementary material for: Audio Recording Patient-Nurse Verbal Communications in Home Health Care Settings: Pilot Feasibility and Usability Study
Source: JMIR Hum Factors. 2022 May 11;9(2):e35325. doi: 10.2196/35325 (PMC9133990; doi:10.2196/35325)
Supplement: Multimedia Appendix 1 [file humanfactors_v9i2e35325_app1.docx]

**Appendix A: Questions for Semi-Structured Interviews**

**Nurse semi-structured interview guide questions:**

1. How many years have you worked as a nurse?
2. Are you Hispanic?
3. What is your race?
4. What is your gender?
5. What is the level of your education?
6. Please describe your experience with recording patient encounters?
7. Does the recording process fit into the homecare workflow?
   1. If yes, how?
   2. If no, why not?
   3. What can be changed about the recording process, if at all?
8. What are characteristics of patients who would be more likely to agree to recording the encounter? What are characteristics of patients who would be more likely to disagree to recording the encounter?
   1. Why?
9. How have your patients and their caregivers responded to the recording process?
10. Does the fact that an encounter is recorded affect patient or caregiver communication with you? Please explain how.
11. Does the fact that an encounter is recorded affect nurses’ communication with their patients and caregivers? Please explain how.
12. Are there any other ways in which the recordings could be useful in your work?
    1. Have you used them for paperwork/notes at the end of the visit?

**Patient semi-structured interview guide questions:**

1. Are you of Hispanic or Latino descent?
2. How would you describe your race?
3. How many years of school were you able to complete/what was the grade you last completed?
4. What was your motivation for having your visits recorded? (why did you decide to allow your nurse to audio record your conversation with him/her)
   1. Did you feel expected to?
   2. Monetary incentive?
   3. It would be beneficial, how?
5. Walk me through the recording process:
   1. Does the clinician ask to record every time?
   2. Are you aware when the recording begins?
   3. Do you ever ask for the recording to be turned off during a visit? i.e., a family member walks in, you get an important pho ne call etc.
6. How do you feel about the recording process?
7. Does the recording affect how you speak with your nurse?
   1. Are you more open or closed?
   2. During the visit, how aware are you of the recording?
   3. Do you think that the recording affects the way the nurse was speaking to you?
